# Correction: Tang et al. Treadmill Exercise Alleviates Cognition Disorder by Activating the FNDC5: Dual Role of Integrin αV/β5 in Parkinson’s Disease. *Int. J. Mol. Sci.* 2023, *24*, 7830

**DOI:** 10.3390/ijms27083577

**Published:** 2026-04-17

**Authors:** Chuanxi Tang, Mengting Liu, Zihang Zhou, Hao Li, Chenglin Yang, Li Yang, Jie Xiang

**Affiliations:** 1 Xuzhou Key Laboratory of Neurobiology, Department of Neurobiology, Xuzhou Medical University, Xuzhou 221004, China202126011208@stu.xzhmu.edu.cn (L.Y.); 2 Department of Rehabilitation, The Affiliated Hospital of Xuzhou Medical University, 99 West Huaihai Road, Xuzhou 221002, China; 3 The College of Medical Technology, Xuzhou Medical University, Xuzhou 221004, China

## Error in Figure

In the original publication [1], there was a mistake in Figure 7A as published. The image was incorrectly hyperlinked during the grouping process. The corrected Figure 7A appears below.

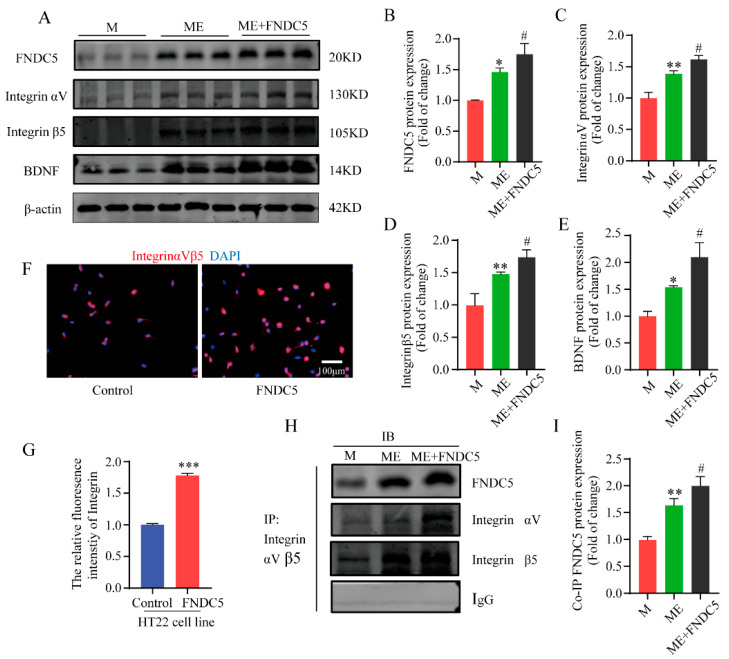



The authors state that the scientific conclusions are unaffected. This correction was approved by the Academic Editor. The original publication has also been updated.
